# A combined intervention of emotional freedom techniques and psychological nursing for reducing anticipatory grief in breast cancer patients undergoing chemotherapy: a retrospective cohort study

**DOI:** 10.3389/fpubh.2026.1876981

**Published:** 2026-07-27

**Authors:** Lili Zhao, Meng Lv

**Affiliations:** 1 Department of Breast Medicine, Affiliated Cancer Hospital of Harbin Medical University, Harbin, China; 2 Thyroid Surgery Department, Breast Disease Diagnosis and Treatment Center, Jinan Central Hospital, Jinan, China

**Keywords:** anticipatory grief, breast cancer chemotherapy, combined intervention, emotional freedom techniques, psychological nursing, retrospective study

## Abstract

**Objective:**

To examine the association between Emotional Freedom Techniques (EFT) combined with psychological nursing and anticipatory grief-related psychological outcomes in breast cancer patients undergoing chemotherapy, and to provide real-world evidence for clinical psychological nursing practice.

**Methods:**

A retrospective cohort study design was adopted, enrolling 158 breast cancer patients who received chemotherapy in our hospital from August 2023 to December 2024. Propensity score matching (PSM) was performed using a 1:1 nearest-neighbor matching method with a caliper width of 0.2 standard deviations of the logit of the propensity score. The matched variables included age, cancer stage, chemotherapy-related clinical characteristics, comorbidities, and baseline psychological assessment scores. After matching, 79 patients were retained in each cohort: a combined-intervention cohort (EFT plus psychological nursing, *n* = 79) and a conventional-nursing cohort (*n* = 79). Because the intervention was a behavioral and psychological nursing intervention, patients and intervention providers could not be blinded. Therefore, this study did not use a true double-blind design. To reduce assessment and analytical bias, outcome assessors who were not involved in intervention delivery reviewed the psychological assessment data, and statistical analysis was performed using coded group labels. The primary outcome was the post-intervention total score of the Preparatory Grief in Advanced Cancer Patients (PGAC) scale. Secondary outcomes included post-intervention anxiety and depression scores measured by the Self-Rating Anxiety Scale (SAS) and Self-Rating Depression Scale (SDS), psychological resilience dimension scores, hope level measured by the Herth Hope Index (HHI), coping strategies measured by the Medical Coping Modes Questionnaire (MCMQ), cancer-related fatigue measured by the Piper Fatigue Scale (PFS), and quality of life measured by the Quality of Life Questionnaire (QoLQ).

**Results:**

After PSM, baseline clinical characteristics and pre-intervention psychological scores were balanced between the two matched cohorts, with all SMDs <0.10. For the predefined primary outcome, the post-intervention PGAC total score was lower in the combined-intervention cohort than in the conventional-nursing cohort (40.56 ± 2.17 vs. 58.94 ± 3.37; MD = −18.38, 95% CI: −19.27 to −17.49; Cohen's d = −6.48; *P* < 0.001). Secondary analyses showed lower post-intervention anxiety, depression, avoidance, surrender, and fatigue scores, as well as higher psychological resilience, hope, confrontation, and quality-of-life scores in the combined-intervention cohort. *P* values, MDs, 95% CIs, and Cohen's d values are reported in the corresponding Results subsections.

**Conclusion:**

In this retrospective cohort study, receipt of EFT combined with psychological nursing was associated with lower anticipatory grief scores and more favorable psychological outcomes among breast cancer patients undergoing chemotherapy. These associations may be related to improved psychological resilience, higher hope levels, and more adaptive coping strategies. The combined program may represent a potentially useful psychological nursing approach for the comprehensive care of breast cancer patients, although causal effects require confirmation in prospective randomized studies.

## 1 Introduction

Breast cancer remains the most prevalent malignant tumor among women globally, with its incidence showing a sustained upward trend. According to the latest data from the International Agency for Research on Cancer (IARC), the global number of newly diagnosed breast cancer cases reached 2.8 million in 2024, accounting for 31.4% of all female malignant tumor diagnoses. Statistics from China's National Cancer Center indicate that while the 5-year survival rate for breast cancer in China has risen to 83.2%, the incidence of psychological complications during treatment remains as high as 76.3%, emerging as a critical factor affecting patients' quality of life (1–4). In the comprehensive treatment framework for breast cancer, chemotherapy serves as a pivotal modality, yet its associated side effects—such as alopecia, nausea, and fatigue—often trigger excessive worry about disease progression and mortality. This psychological response is defined as “anticipatory grief” in medical psychology. Anticipatory grief represents a unique psychological stress reaction among cancer patients, characterized by fear of treatment-related adverse effects, sadness related to loss of role function, and anxiety about future uncertainties (5, 6). Literature reports indicate that anticipatory grief occurs in 68–82% of breast cancer patients undergoing chemotherapy and is significantly associated with reduced treatment adherence, deteriorated quality of life, and shortened survival duration (7–9). A meta-analysis incorporating 12 studies revealed that unaddressed anticipatory grief increases the risk of chemotherapy interruption by 2.3-fold and elevates the 5-year recurrence rate by 1.8-fold. This psychological state not only disrupts treatment progression but may also establish a vicious cycle of “psychological stress-physiological response-disease progression”.

Retrospective cohort designs offer advantages in rapidly acquiring clinical data and reflecting real-world clinical practice, but they are primarily suitable for examining associations rather than establishing causal effects. This study enrolled 158 breast cancer patients receiving chemotherapy between August 2023 and December 2024. Using propensity score matching (PSM) to reduce baseline imbalance, we compared psychological and quality-of-life outcomes between patients who received EFT combined with psychological nursing and those who received conventional nursing. Multidimensional assessment tools were used to examine differences in psychological state, coping strategies, and quality of life between the two cohorts.

## 2 Materials and methods

### 2.1 Study population

This study was designed as a retrospective cohort study and included breast cancer patients who received chemotherapy at our hospital between August 2023 and December 2024. After eligibility screening, 158 patients were included in the matched cohort analysis. Eligible patients were first classified according to documented nursing exposure into a combined-intervention cohort and a conventional-nursing cohort. PSM was then performed to reduce baseline imbalance between the two cohorts. After 1:1 matching, 79 patients were retained in the combined-intervention cohort (EFT plus psychological nursing) and 79 patients were retained in the conventional-nursing cohort for comparison of psychological and quality-of-life outcomes. This retrospective cohort study involving human participants was reviewed and approved by the Ethics Committee of Jinan Central Hospital (Approval No.: FKLC23HL02; Approval date: 2023-06-11). The study was conducted in accordance with the Declaration of Helsinki and relevant institutional and national regulations on medical data protection. Because this study used only anonymized existing clinical records and psychological assessment data collected during routine care, involved no additional intervention, examination, follow-up procedure, or risk to participants, and did not include identifiable patient images or personal information, the Ethics Committee of Jinan Central Hospital waived the requirement for additional study-specific informed consent. Patient privacy was protected by de-identifying all data before analysis, removing personal identifiers such as names and identification numbers, restricting access to the dataset to authorized researchers only, and presenting all results in aggregate form to prevent individual identification.

### 2.2 Inclusion and exclusion criteria

Inclusion criteria: pathologically confirmed breast cancer diagnosis; age ≥30 years; complete clinical records; complete baseline and post-intervention psychological assessment records; normal cognitive function enabling independent completion of assessments; and receipt of standardized chemotherapy at our hospital (10, 11). Exclusion criteria: history of other malignancies; significant dysfunction of vital organs (liver, kidney, brain); presence of psychiatric disorders or impaired consciousness; long-term use of anxiolytic or antidepressant medications; incomplete clinical records; missing baseline or post-intervention psychological assessment data; or withdrawal from psychological assessment during the observation period.

### 2.3 Propensity score matching

To reduce potential selection bias and baseline imbalance inherent to the retrospective cohort design, propensity score matching was performed before outcome comparisons. The propensity score was estimated using a binary logistic regression model, with receipt of EFT combined with psychological nursing as the dependent variable. Clinically relevant variables that could influence both nursing exposure and psychological outcomes were included in the propensity score model, including age, BMI, tumor stage, molecular subtype, treatment status, chemotherapy regimen, surgery before chemotherapy, radiotherapy history, endocrine therapy, comorbidities, and baseline psychological assessment scores, including baseline SAS, SDS, PGAC, HHI, MCMQ, PFS, and QoLQ scores. A 1:1 nearest-neighbor matching method without replacement was used, with a caliper width of 0.2 standard deviations of the logit of the propensity score. After matching, 79 patients in the combined-intervention cohort and 79 patients in the conventional-nursing cohort were retained.

Covariate balance before and after matching was assessed using standardized mean differences (SMDs). An SMD of less than 0.10 was considered to indicate acceptable balance between the two cohorts. In addition, baseline characteristics and pre-intervention psychological scores were compared after matching using appropriate statistical tests. After PSM, no clinically meaningful imbalance remained between the two cohorts, supporting the comparability of the matched study population.

### 2.4 Intervention protocols

All nursing interventions were delivered during the chemotherapy period. The conventional-nursing cohort received routine chemotherapy-related nursing care, whereas the combined-intervention cohort received EFT combined with structured psychological nursing in addition to routine nursing care. The intervention lasted for 2 consecutive weeks. All intervention procedures were documented in the nursing records, including the date, duration, intervention content, patient participation, and any discomfort or adverse response.

#### 2.4.1 Conventional nursing

Patients in the conventional-nursing cohort received routine chemotherapy nursing care. This included pre-chemotherapy preparation, explanation of the chemotherapy process, treatment-related health education, assistance with examinations, monitoring of vital signs and adverse reactions, and timely feedback of abnormal symptoms to the medical team. Nurses provided guidance on diet, sleep, activity, and management of common chemotherapy-related symptoms, including nausea, vomiting, fatigue, appetite loss, constipation, urinary discomfort, skin reactions, and local infusion-site irritation. Patients were encouraged to maintain moderate daily activity according to their physical condition. Before discharge or treatment interval, patients received home-care instructions and a 24-h contact number for treatment-related questions.

#### 2.4.2 Combined intervention: EFT plus psychological nursing

Patients in the combined-intervention cohort received the above routine nursing care plus EFT and structured psychological nursing. The intervention was implemented by oncology nurses who had received standardized training. The training covered the basic principles of EFT, acupoint localization, communication skills, psychological risk identification, intervention documentation, and emergency handling of emotional distress. Before delivering the intervention independently, nurses were required to complete a competency assessment conducted by the head nurse and the psychological nursing coordinator.

#### 2.4.3 EFT Protocol

EFT was performed in a quiet consultation room or bedside environment with adequate privacy. Each session lasted approximately 15–20 min and was conducted twice daily, once in the morning and once in the evening, for 2 consecutive weeks. Before each session, the nurse asked the patient to identify the main negative emotion or concern at that moment, such as fear of chemotherapy, worry about disease progression, body image distress, fatigue, sleep disturbance, or uncertainty about prognosis. The patient then rated the intensity of distress using a 0–10 subjective distress score, with 0 indicating no distress and 10 indicating the strongest distress.

Each EFT session consisted of four steps. First, the nurse guided the patient to perform slow breathing for 1–2 min and helped the patient formulate an individualized acceptance statement. A typical statement was: “Although I feel afraid and worried about chemotherapy, I am willing to accept my current feelings and face treatment step by step.” The statement could be adjusted according to the patient's main concern. Second, the nurse guided the patient to tap the selected acupoints using the index, middle, and ring fingers together with moderate force. The tapping sequence included Houxi (SI3), Neiguan (PC6), Cuanzhu (BL2), Tongziliao (GB1), Chengqi (ST1), Chengjiang (CV24), Shenzang (KI15), Dabao (SP21), and Baihui (GV20). Each acupoint was tapped approximately 5–7 times, and the whole sequence was repeated for 3 cycles. Third, during tapping, the patient was encouraged to repeat short positive phrases, such as “I am safe at this moment,” “I can cooperate with treatment,” “My body is receiving care,” or “I can face this process gradually.” Fourth, after tapping, the patient completed 3 cycles of diaphragmatic breathing. The subjective distress score was reassessed at the end of the session. If the score remained high, the nurse provided additional emotional support and recorded the patient's response.

Eye movement integration was used as a supplementary component after acupoint tapping. Patients were instructed to perform horizontal eye movements, vertical eye movements, clockwise and counterclockwise eye rotation, and brief humming of a familiar or preferred song. Each movement was performed slowly and comfortably, and the full sequence was repeated 3 times. If dizziness, eye discomfort, nausea, or obvious fatigue occurred, the exercise was stopped immediately and recorded.

#### 2.4.4 Structured psychological nursing

Structured psychological nursing was delivered through four components: individualized emotional support, mindfulness relaxation, group psychological support, and family involvement.

First, individualized emotional support was provided by the responsible nurse at least twice weekly, with each session lasting approximately 20–30 min. Additional brief bedside communication was provided when patients showed obvious anxiety, sadness, fear, irritability, or avoidance. The nurse used open-ended questions to identify the patient's main psychological concerns, such as fear of recurrence, chemotherapy-related adverse reactions, changes in body image, family burden, financial stress, and uncertainty about prognosis. Nurses provided disease-related explanations using simple and non-threatening language, corrected misunderstandings about chemotherapy, and helped patients set short-term achievable goals, such as completing the current chemotherapy cycle, maintaining food intake, improving sleep, or communicating actively with family members.

Second, mindfulness relaxation was conducted once daily for approximately 15 min. Patients were guided to sit or lie in a comfortable position in a quiet environment. The nurse played soft background music when appropriate and guided patients to focus on slow thoracoabdominal breathing. Patients were then instructed to observe bodily sensations without judgment and to relax the head, shoulders, chest, abdomen, limbs, and feet sequentially. At the end of the exercise, the nurse encouraged patients to describe their current emotional state and any change in physical discomfort.

Third, group psychological support was organized in small groups of 5–8 patients when patient condition and treatment schedule allowed. Six themed sessions were conducted over 2 weeks, with each session lasting approximately 45–60 min. The themes were as follows: Session 1, “Getting Acquainted,” focusing on ice-breaking and establishing group trust; Session 2, “Emotion Expression,” encouraging patients to express fear, sadness, and treatment-related stress; Session 3, “Self-Awareness,” guiding patients to recognize negative thoughts and reconstruct more adaptive interpretations; Session 4, “Social Support,” helping patients identify available family, peer, and medical support resources; Session 5, “Positive Outlook,” encouraging patients to establish short-term treatment and life goals; and Session 6, “Future Messages,” asking patients to write encouraging messages to themselves or other patients to consolidate positive expectations.

Fourth, family involvement was encouraged when appropriate. Nurses communicated with family members to explain the patient's possible emotional reactions during chemotherapy and encouraged family members to provide supportive companionship, avoid excessive negative language, and assist patients in maintaining daily routines. Family members were also encouraged to reinforce the patient's short-term goals and provide practical support during chemotherapy intervals.

#### 2.4.5 Intervention fidelity and documentation

To improve consistency, all nurses followed the same intervention checklist. The checklist included session date, session duration, EFT completion, distress score before and after EFT, acupoints tapped, mindfulness completion, psychological nursing content, group session attendance, patient cooperation, and adverse responses. The head nurse reviewed the intervention records weekly. Missed sessions due to examination, fatigue, or temporary discomfort were rescheduled within the same week when possible. If a patient developed severe emotional distress, suicidal ideation, or psychiatric symptoms, the nurse was required to stop the session and refer the patient to the physician or mental health specialist immediately.

### 2.5 Outcomes and observational indicators

Blinding was handled at the outcome assessment and statistical analysis levels rather than at the patient or intervention-provider level. Because EFT combined with psychological nursing required direct patient participation and face-to-face implementation by trained nursing staff, blinding of patients and intervention providers was not feasible. To reduce potential assessment bias, psychological assessment data were reviewed by trained assessors who were not involved in the delivery of the intervention. During statistical analysis, group labels were coded so that the statistician was not informed of the specific cohort identity until the main analyses were completed.

The outcome framework was defined before outcome comparison. The primary outcome was the post-intervention total PGAC score, which was selected because anticipatory grief was the main target of the study. Baseline PGAC score was used as a matching and balance variable rather than as the primary endpoint. The primary comparison was the between-cohort difference in post-intervention PGAC total score after propensity score matching.

Secondary outcomes included post-intervention SAS and SDS scores, psychological resilience dimension scores, HHI score, MCMQ confrontation, avoidance, and surrender scores, PFS dimension scores, and QoLQ functional domain scores. These secondary outcomes were used to provide a broader evaluation of emotional distress, psychological adjustment, coping style, fatigue, and quality of life. Baseline psychological scores were reported in Table 1 to demonstrate post-matching comparability between cohorts, whereas post-intervention outcomes were reported in the Results section and figures. For all included patients, psychological assessments were required to be available at both baseline and after the intervention period. The completeness of PGAC, SAS, SDS, HHI, MCMQ, PFS, and QoLQ records was checked before propensity score matching. Patients with missing scale items, incomplete questionnaires, or unavailable post-intervention psychological assessment records were excluded before matching. Therefore, all 158 patients retained in the final matched cohort completed all prespecified baseline and post-intervention psychological assessments.

**Table 1:** Baseline characteristics of the two cohorts after propensity score matching.

Characteristic	Experimental group	Control group	*t*/x^2^	*P*
Number of cases	79	79		
Age, years	47.94 ± 5.28	48.24 ± 5.36	0.724	0.056
BMI, kg/m^2^	23.18 ± 2.36	23.31 ± 2.42	0.733	0.054
Tumor stage			0.945	0.084
Stage I	23 (29.1)	25 (31.6)		
Stage II	26 (32.9)	25 (31.6)		
Stage III	21 (26.6)	22 (27.8)		
Stage IV	9 (11.4)	7 (8.9)		
Molecular subtype			0.991	0.033
Luminal A	18 (22.8)	19 (24.1)		
Luminal B	29 (36.7)	28 (35.4)		
HER2–enriched	15 (19.0)	14 (17.7)		
Triple-negative	17 (21.5)	18 (22.8)		
Treatment status			0.828	0.084
Neoadjuvant chemotherapy	31 (39.2)	30 (38.0)		
Adjuvant chemotherapy	39 (49.4)	42 (53.2)		
Palliative chemotherapy	9 (11.4)	7 (8.9)		
Chemotherapy regimen			0.990	0.052
AC-T regimen	30 (38.0)	32 (40.5)		
TC regimen	24 (30.4)	23 (29.1)		
EC-T regimen	16 (20.3)	15 (19.0)		
Other regimens	9 (11.4)	9 (11.4)		
Surgery before chemotherapy	39 (49.4)	42 (53.2)	0.633	0.076
Previous or concurrent radiotherapy	11 (13.9)	12 (15.2)	0.822	0.036
Endocrine therapy	28 (35.4)	30 (38.0)	0.741	0.053
At least one comorbidity	25 (31.6)	26 (32.9)	0.865	0.027
Hypertension	15 (19.0)	14 (17.7)	0.837	0.033
Diabetes mellitus	8 (10.1)	9 (11.4)	0.797	0.041
Cardiovascular disease	5 (6.3)	6 (7.6)	0.755	0.050
Baseline SAS score	70.12 ± 5.63	70.39 ± 5.71	0.765	0.048
Baseline SDS score	73.10 ± 6.04	72.86 ± 6.18	0.805	0.039
Baseline PGAC score	60.21 ± 3.44	60.48 ± 3.39	0.620	0.079
Baseline HHI score	16.32 ± 1.82	16.47 ± 1.88	0.611	0.081
Baseline MCMQ confrontation score	20.43 ± 2.31	20.20 ± 2.38	0.539	0.098
Baseline MCMQ avoidance score	12.36 ± 1.92	12.51 ± 1.95	0.627	0.078
Baseline MCMQ surrender score	8.37 ± 1.23	8.46 ± 1.28	0.653	0.072
Baseline PFS total score	113.52 ± 7.86	113.06 ± 8.02	0.716	0.058
Baseline QoLQ total score	60.14 ± 5.48	59.75 ± 5.62	0.659	0.070

Values are presented as mean ± standard deviation or *n* (%). *P* values were calculated using the independent-samples t test for continuous variables and the chi-square test or Fisher's exact test for categorical variables, as appropriate. PSM was performed using 1:1 nearest-neighbor matching without replacement, with a caliper width of 0.2 standard deviations of the logit of the propensity score. Balance was assessed using standardized mean differences (SMDs), and an SMD < 0.10 was considered to indicate acceptable balance. For multi-category variables, the reported SMD represents the maximum category-specific SMD.

This study employed a multidimensional psychological assessment system covering seven core domains:

Negative Emotions: Assessed via Self-Rating Anxiety Scale (SAS) and Self-Rating Depression Scale (SDS). A score of 50 was used as the cutoff for SAS, with scores of 50–59, 60–69, and ≥70 indicating mild, moderate, and severe anxiety, respectively. A score of 53 was used as the cutoff for SDS, with scores of 53–62, 63–72, and ≥73 indicating mild, moderate, and severe depression, respectively. Higher scores indicate greater emotional distress.

Psychological Resilience: Psychological resilience was measured using a three-dimensional scale covering tenacity (13 items), strength (8 items), and optimism (4 items), with each item scored from 0 to 4. Total scores reflected psychological adjustment capacity.

Coping Styles: Evaluated through Medical Coping Modes Questionnaire (MCMQ) covering surrender (5 items), avoidance (7 items), and confrontation (8 items) dimensions. A 4-point Likert scale quantified coping tendencies.

Hope Levels: Assessed using Herth Hope Index (HHI) with 12 items (1–4 points each), total scores ranging 12–48. Higher scores indicate higher levels of hope.

Anticipatory Grief: Measured by the Chinese-validated Preparatory Grief in Advanced Cancer Patients (PGAC) scale (CVI = 0.916, Cronbach's α = 0.919). The 31-item instrument covered 7 dimensions with 0–3 point scoring, higher total scores indicate greater grief severity.

Cancer-Related Fatigue: Assessed via Piper Fatigue Scale (PFS) covering behavioral, emotional, perceptual, and cognitive dimensions (22 items total). Each item used 0- 10 point scoring, with lower scores indicating milder fatigue.

Quality of Life: Evaluated through the self-administered QoLQ scale covering physiological, physical, social, and psychological functional domains. Higher scores indicate better quality of life.

### 2.6 Statistical analysis

Image processing was conducted using GraphPad Prism 8. Data organization and analysis were performed with SPSS 26.0. Quantitative data were expressed as mean ± standard deviation, and categorical data were presented as *n* (%). The statistical analysis was organized according to the predefined outcome hierarchy. The primary analysis compared the post-intervention PGAC total score between the combined-intervention cohort and the conventional-nursing cohort after propensity score matching. Secondary analyses compared post-intervention SAS, SDS, psychological resilience, HHI, MCMQ, PFS, and QoLQ scores between the two matched cohorts.

For continuous outcomes, between-cohort mean differences (MDs), 95% confidence intervals (CIs), *P* values, and Cohen's d values were calculated. MDs were calculated as the mean value in the combined-intervention cohort minus the mean value in the conventional-nursing cohort. Cohen's d was calculated using the pooled standard deviation, with positive values indicating higher scores in the combined-intervention cohort and negative values indicating lower scores in the combined-intervention cohort. Exact *P* values were reported when *P*≥0.001, whereas values smaller than 0.001 were reported as *P* < 0.001 to avoid overprecision. For categorical variables, the chi-square test or Fisher's exact test was used, as appropriate. Standardized mean differences (SMDs) were used to evaluate baseline balance after propensity score matching. A two-sided *P* value < 0.05 was considered statistically significant for the primary outcome, whereas secondary outcomes were interpreted as supportive analyses. *P* values, MDs, 95% CIs, and Cohen's d values are reported in the corresponding Results subsections. Missing data were handled before propensity score matching. Patients with incomplete clinical variables required for the propensity score model or missing baseline or post-intervention psychological assessment data were excluded during eligibility screening. No statistical imputation was performed because the final matched dataset contained complete clinical and psychological assessment data for all analyzed patients. Therefore, all outcome analyses were conducted using a complete-case approach.

## 3 Results

### 3.1 Baseline characteristics

After PSM, 158 patients were included in the matched analysis, with 79 patients in the combined-intervention cohort and 79 patients in the conventional-nursing cohort. All patients retained in the matched cohort had complete clinical variables for PSM and complete baseline and post-intervention psychological assessment records for PGAC, SAS, SDS, HHI, MCMQ, PFS, and QoLQ. The two matched cohorts were comparable in demographic characteristics, tumor-related clinical variables, treatment status, chemotherapy regimen, comorbidities, and baseline psychological assessment scores. All included baseline variables achieved acceptable balance after matching, with standardized mean differences (SMDs) below 0.10. See Table 1.

### 3.2 Secondary outcome: negative emotions

**Figure 1:**
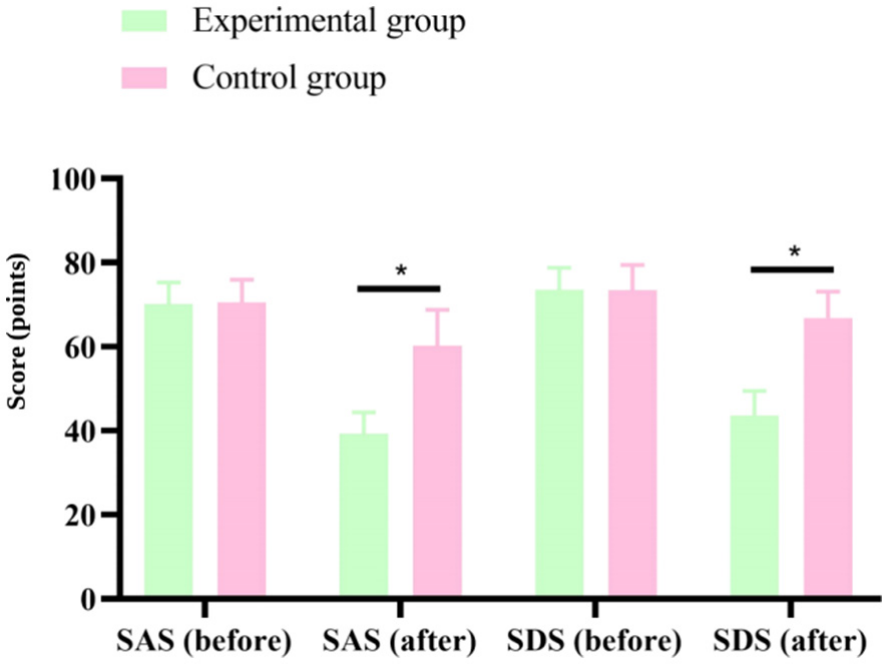
SAS and SDS scores before and after the intervention period in the two matched cohorts. Data are presented as mean ± standard deviation, and error bars represent standard deviations. The combined-intervention cohort included 79 patients, and the conventional-nursing cohort included 79 patients. Between-cohort comparisons of post-intervention scores were performed using the independent-samples *t* test. Detailed P values, mean differences, 95% confidence intervals, and Cohen's d values are reported in the corresponding Results subsection. SAS, Self-Rating Anxiety Scale; SDS, Self-Rating Depression Scale.

Baseline SAS and SDS scores were balanced between the two matched cohorts after PSM (SAS: *P* = 0.765, SMD = 0.048; SDS: *P* = 0.805, SMD = 0.039). After the intervention period, lower SAS and SDS scores were observed in the combined-intervention cohort than in the conventional-nursing cohort. For SAS, the combined-intervention cohort had a lower score than the conventional-nursing cohort (39.25 ± 5.14 vs. 60.25 ± 8.55; MD = −21.00, 95% CI: −23.22 to −18.78; Cohen's d = −2.98; *P* < 0.001). For SDS, the combined-intervention cohort also had a lower score than the conventional-nursing cohort (43.65 ± 5.78 vs. 66.87 ± 6.24; MD = −23.22, 95% CI: −25.11 to −21.33; Cohen's d = −3.86; *P* < 0.001). See Figure 1.

### 3.3 Secondary outcome: psychological resilience

**Figure 2:**
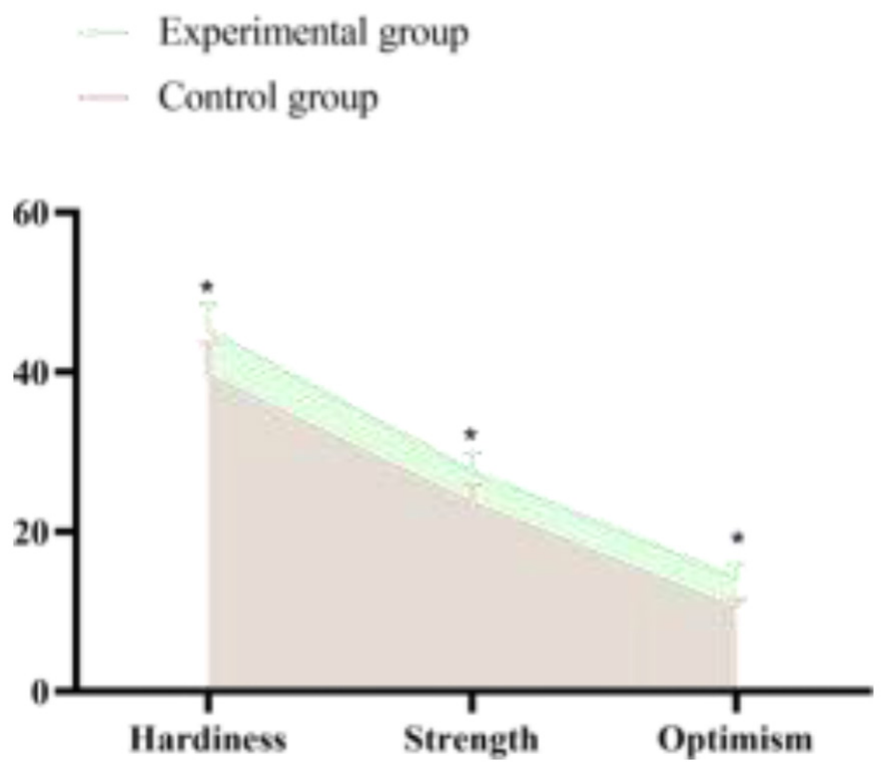
Psychological resilience dimension scores after the intervention period in the two matched cohorts. Data are presented as mean ± standard deviation, and error bars represent standard deviations. The combined-intervention cohort included 79 patients, and the conventional-nursing cohort included 79 patients. Between-cohort comparisons were performed using the independent-samples *t* test. Detailed *P* values, mean differences, 95% confidence intervals, and Cohen's d values are reported in the corresponding Results subsection.

After the intervention period, higher scores for all psychological resilience dimensions were observed in the combined-intervention cohort than in the conventional-nursing cohort. The between-cohort differences were as follows: tenacity, 45.25 ± 3.25 vs. 39.45 ± 4.11, MD = 5.80, 95% CI: 4.64 to 6.96, Cohen's d = 1.57, *P* < 0.001; strength, 27.58 ± 2.28 vs. 23.58 ± 2.25, MD = 4.00, 95% CI: 3.29 to 4.71, Cohen's d = 1.77, *P* < 0.001; and optimism, 14.19 ± 1.77 vs. 10.36 ± 1.16, MD = 3.83, 95% CI: 3.36 to 4.30, Cohen's d = 2.56, *P* < 0.001. See Figure 2.

### 3.4 Secondary outcome: coping strategies

**Figure 3:**
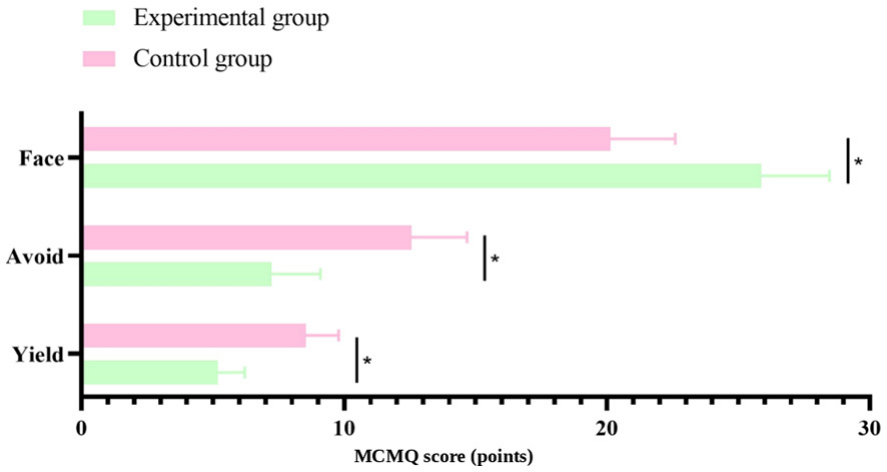
MCMQ dimension scores after the intervention period in the two matched cohorts. Data are presented as mean ± standard deviation, and error bars represent standard deviations. The combined-intervention cohort included 79 patients, and the conventional-nursing cohort included 79 patients. Between-cohort comparisons were performed using the independent-samples *t* test. Detailed *P* values, mean differences, 95% confidence intervals, and Cohen's d values are reported in the corresponding Results subsection. MCMQ, Medical Coping Modes Questionnaire.

After the intervention period, lower MCMQ scores for surrender and avoidance and a higher score for confrontation were observed in the combined-intervention cohort than in the conventional-nursing cohort. The between-cohort differences were as follows: surrender, 5.21 ± 1.01 vs. 8.54 ± 1.25, MD = −3.33, 95% CI: −3.69 to −2.97, Cohen's d = −2.93, *P* < 0.001; avoidance, 7.25 ± 1.85 vs. 12.56 ± 2.11, MD = −5.31, 95% CI: −5.93 to −4.69, Cohen's d = −2.68, *P* < 0.001; and confrontation, 25.88 ± 2.58 vs. 20.13 ± 2.47, MD = 5.75, 95% CI: 4.96 to 6.54, Cohen's d = 2.28, *P* < 0.001. See Figure 3.

### 3.5 Secondary outcome: hope levels

**Figure 4:**
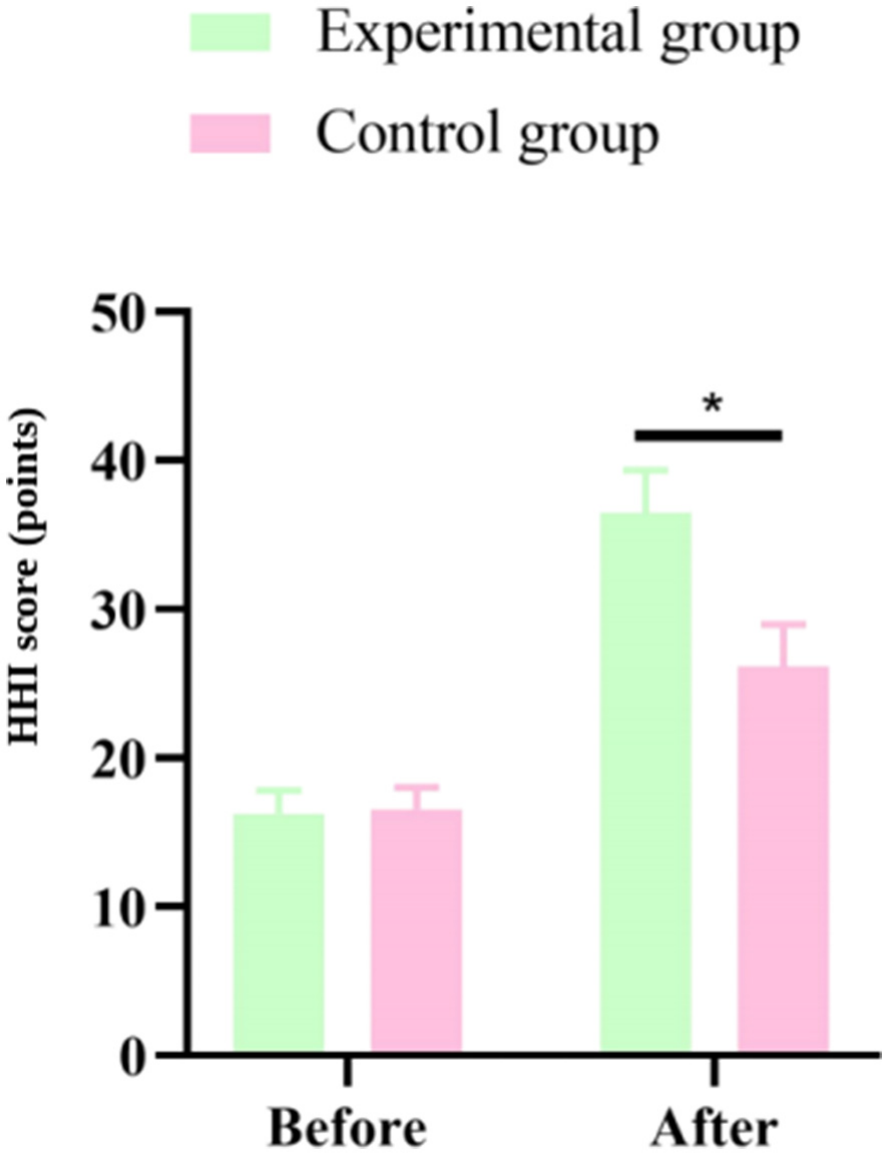
HHI scores before and after the intervention period in the two matched cohorts. Data are presented as mean ± standard deviation, and error bars represent standard deviations. The combined-intervention cohort included 79 patients, and the conventional-nursing cohort included 79 patients. Between-cohort comparisons of post-intervention scores were performed using the independent-samples *t* test. Detailed *P* values, mean differences, 95% confidence intervals, and Cohen's d values are reported in the corresponding Results subsection. HHI, Herth Hope Index.

Baseline HHI scores were balanced between the two matched cohorts after PSM (*P* = 0.611, SMD = 0.081). After the intervention period, higher HHI scores were observed in the combined-intervention cohort than in the conventional-nursing cohort (36.48 ± 2.89 vs. 26.14 ± 2.84; MD = 10.34, 95% CI: 9.44 to 11.24; Cohen's d = 3.61; *P* < 0.001). See Figure 4.

### 3.6 Primary outcome: anticipatory grief

**Figure 5:**
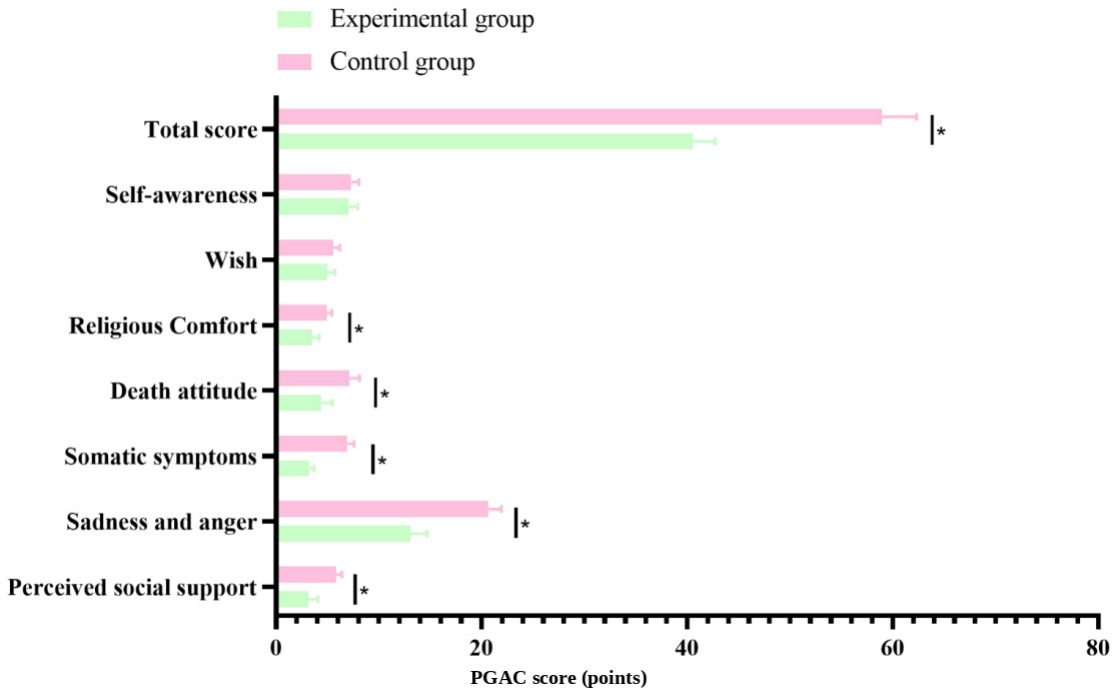
PGAC total and dimension scores after the intervention period in the two matched cohorts. Data are presented as mean ± standard deviation, and error bars represent standard deviations. The combined-intervention cohort included 79 patients, and the conventional-nursing cohort included 79 patients. Between-cohort comparisons were performed using the independent-samples *t* test. The PGAC total score was the predefined primary outcome. Detailed *P* values, mean differences, 95% confidence intervals, and Cohen's d values for the PGAC total score are reported in the corresponding Results subsection. PGAC, Preparatory Grief in Advanced Cancer Patients.

For the predefined primary outcome, lower post-intervention PGAC total scores were observed in the combined-intervention cohort than in the conventional-nursing cohort (40.56 ± 2.17 vs. 58.94 ± 3.37). The MD was −18.38 (95% CI: −19.27 to −17.49), with a Cohen's d of −6.48 and *P* < 0.001. See Figure 5.

### 3.7 Secondary outcome: cancer-related fatigue

**Figure 6:**
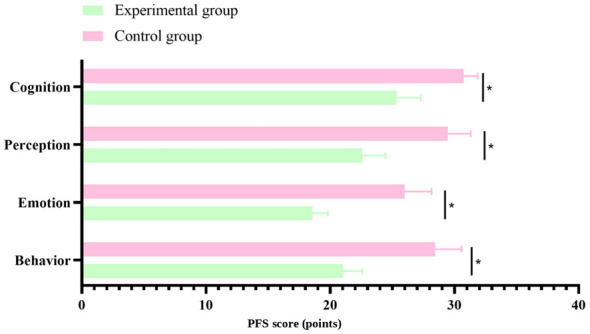
PFS dimension scores after the intervention period in the two matched cohorts. Data are presented as mean ± standard deviation, and error bars represent standard deviations. The combined-intervention cohort included 79 patients, and the conventional-nursing cohort included 79 patients. Between-cohort comparisons were performed using the independent-samples *t* test. Detailed *P* values, mean differences, 95% confidence intervals, and Cohen's d values are reported in the corresponding Results subsection. PFS, Piper Fatigue Scale.

After the intervention period, lower scores for all PFS dimensions were observed in the combined-intervention cohort than in the conventional-nursing cohort. The between-cohort differences were as follows: behavioral fatigue, 21.02 ± 1.56 vs. 28.45 ± 2.11, MD = −7.43, 95% CI: −8.01 to −6.85, Cohen's d = −4.00, *P* < 0.001; emotional fatigue, 18.56 ± 1.25 vs. 25.98 ± 2.18, MD = −7.42, 95% CI: −7.98 to −6.86, Cohen's d = −4.18, *P* < 0.001; perceptual fatigue, 22.58 ± 1.85 vs. 29.45 ± 1.85, MD = −6.87, 95% CI: −7.45 to −6.29, Cohen's d = −3.71, *P* < 0.001; and cognitive fatigue, 25.35 ± 1.93 vs. 30.74 ± 1.13, MD = −5.39, 95% CI: −5.89 to −4.89, Cohen's d = −3.41, *P* < 0.001. See Figure 6.

### 3.8 Secondary outcome: quality of life

**Figure 7:**
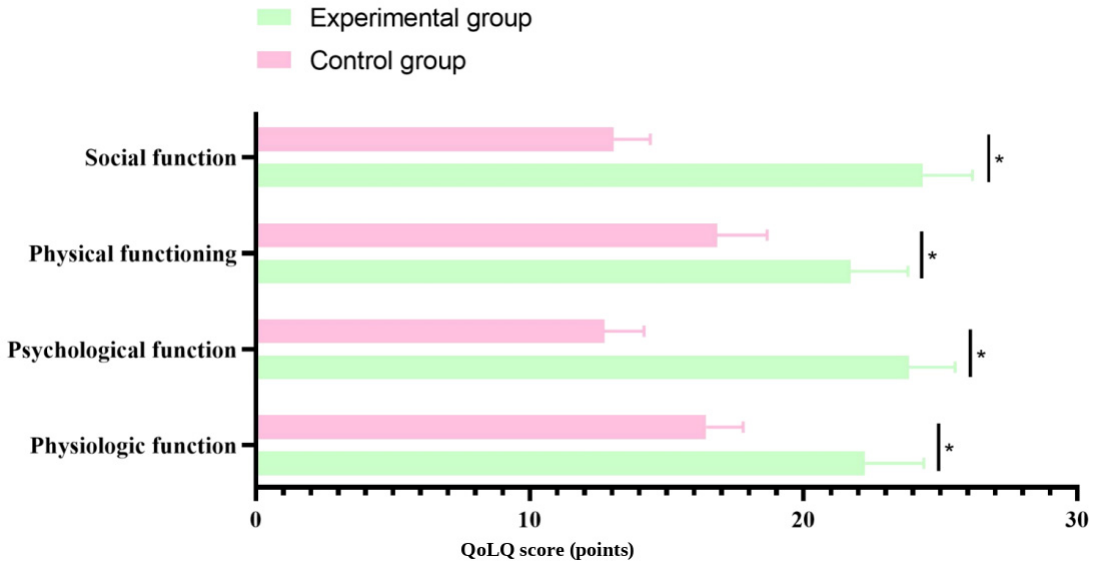
QoLQ functional domain scores after the intervention period in the two matched cohorts. Data are presented as mean ± standard deviation, and error bars represent standard deviations. The combined-intervention cohort included 79 patients, and the conventional-nursing cohort included 79 patients. Between-cohort comparisons were performed using the independent-samples *t* test. Detailed *P* values, mean differences, 95% confidence intervals, and Cohen's d values are reported in the corresponding Results subsection. QoLQ, Quality of Life Questionnaire.

After the intervention period, higher QoLQ scores in all functional domains were observed in the combined-intervention cohort than in the conventional-nursing cohort. The between-cohort differences were as follows: physiological function, 22.25 ± 2.14 vs. 16.44 ± 1.35, MD = 5.81, 95% CI: 5.25 to 6.37, Cohen's d = 3.25, *P* < 0.001; physical function, 23.85 ± 1.69 vs. 12.73 ± 1.44, MD = 11.12, 95% CI: 10.63 to 11.61, Cohen's d = 7.08, *P* < 0.001; social function, 21.73 ± 2.08 vs. 16.85 ± 1.82, MD = 4.88, 95% CI: 4.27 to 5.49, Cohen's d = 2.50, *P* < 0.001; and psychological function, 24.36 ± 1.81 vs. 13.07 ± 1.33, MD = 11.29, 95% CI: 10.79 to 11.79, Cohen's d = 7.11, *P* < 0.001. See Figure 7.

## 4 Discussion

Current clinical interventions primarily include pharmacological anti-anxiety treatments and single-modality psychological interventions. While selective serotonin reuptake inhibitors (SSRIs) can alleviate anxiety symptoms, they carry risks of dependency and drug interactions. Cognitive behavioral therapy (CBT), though proven effective, requires sustained involvement of professional psychotherapists, with implementation rates below 30% in oncology specialty hospitals. Emotional Freedom Techniques (EFT), an emerging somatic-psychological intervention, combines specific acupoint tapping with cognitive restructuring and has demonstrated rapid reduction of stress hormone levels (12, 13). A systematic review incorporating 46 studies revealed that EFT achieves a larger treatment effect size (Cohen's d = 1.23) for anxiety disorders compared to traditional CBT (d = 0.68). However, existing research predominantly focuses on single-modality interventions, lacking systematic evaluation of multimodal combined protocols.

Our team observed in early clinical practice that breast cancer patients undergoing chemotherapy often present with multidimensional psychological and physical concerns, including comorbid anxiety/depression, negative coping styles, and cancer-related fatigue. These problems interact to form complex clinical syndromes, suggesting the need for integrated nursing protocols encompassing emotion regulation, cognitive intervention, and social support (14, 15). Based on these observations, we developed an EFT combined with structured psychological nursing model, aiming to systematically improve psychological resilience and coping capacity through a three-dimensional pathway of “somatic intervention—cognitive restructuring—social support”.

In this retrospective matched cohort, lower PGAC scores were observed in patients who received EFT combined with psychological nursing than in those who received conventional nursing. This finding may be related to improvements in emotional expression, perceived control, treatment-related cognition, and supportive communication during chemotherapy. Previous studies have discussed EFT in relation to acupoint tapping, stress-response regulation, and psychological symptom relief (16, 17). However, the present study did not directly measure neuroendocrine indicators, neurotransmitters, brain structure, or brain network activity. Therefore, possible mechanisms involving cortisol regulation, GABAergic activity, neuroplasticity, the amygdala, or the default mode network should be interpreted only as hypotheses based on previous literature rather than as mechanisms demonstrated by the present study (18–20). The lower SAS and SDS scores observed in the combined-intervention cohort may be associated with repeated emotional labeling, acceptance-based statements, relaxation breathing, and individualized psychological support. Mindfulness relaxation and structured communication may also have helped patients reduce repetitive negative thinking and improve emotional adjustment. Similarly, the higher HHI scores may be related to goal setting, hope-oriented communication, family support, and the construction of short-term achievable treatment goals. These explanations are mainly psychological and behavioral interpretations based on the intervention content and observed scale outcomes, rather than direct biological evidence.

The higher confrontation scores and lower avoidance and surrender scores observed in the combined-intervention cohort may be explained by the structured coping support provided during the intervention. Through repeated emotional identification, acceptance statements, positive self-suggestion, and problem-solving communication, patients may have become more willing to face chemotherapy-related stressors and adopt active coping strategies. Psychological nursing also provided concrete behavioral guidance and social support, which may have improved perceived coping efficacy. The more favorable PFS and QoLQ scores observed in the combined-intervention cohort may be associated with better psychological adjustment, improved coping resources, relaxation practice, and enhanced family or peer support. Previous studies have explored possible relationships between EFT and stress-related biomarkers or inflammatory markers (21, 22). However, this study did not measure TNF-α, IL-6, cortisol, GABA, BDNF, or other biological indicators. Therefore, causal statements suggesting that fatigue or quality-of-life changes were directly mediated by inflammatory cytokines or neurobiological pathways have been removed. The present findings should be interpreted as clinical associations between receipt of the combined intervention and patient-reported psychological and quality-of-life outcomes.

In summary, EFT is a somatic-psychological intervention that combines acupoint tapping, acceptance statements, positive self-suggestion, breathing regulation, and attention-shifting exercises (23, 24). In the present study, EFT was combined with structured psychological nursing, including individualized emotional support, mindfulness relaxation, group psychological support, and family involvement. From a clinical perspective, this combined approach may help patients express negative emotions, restructure treatment-related concerns, enhance perceived support, and adopt more active coping strategies. Previous studies have discussed EFT in relation to cognitive-behavioral, somatic, and attention-regulation processes (25–28). However, because this retrospective cohort study evaluated patient-reported scale outcomes rather than biological or neuroimaging markers, the underlying biological mechanisms remain uncertain. Future prospective studies incorporating biomarkers and neurophysiological assessments are needed to clarify whether neuroendocrine, inflammatory, or brain network changes are involved.

### 4.1 Limitations and future directions

Although this study observed favorable associations between EFT combined with psychological nursing and psychological outcomes in breast cancer patients undergoing chemotherapy, the research design still has the following limitations: First, the retrospective nature of the study inherently makes it susceptible to selection bias. Despite using propensity score matching (PSM) to control baseline differences, potential differences in intervention compliance between the experimental and control groups remain, which may affect the generalizability of results. In addition, because this was a behavioral and psychological nursing intervention, patients and intervention providers could not be blinded. Although outcome assessment and statistical analysis were conducted with blinded or coded procedures where feasible, the absence of full blinding may still have introduced performance or response bias. Future studies need to conduct multi-center randomized controlled trials (RCTs) with random grouping and rigorous quality control to further verify the universality of the intervention protocol.

Second, the observation period of this study only covered the chemotherapy phase and did not include long-term follow-up data after treatment completion. Existing evidence indicates that psychological distress in breast cancer patients may persist into the recovery period, with anxiety recurrence risks remaining even 5 years post-surgery. Therefore, extending follow-up to 6–12 months post-chemotherapy and dynamically monitoring the trajectory of indicators such as anticipatory grief and quality of life is crucial for comprehensively assessing the long-term effects of the intervention.

Third, the absence of biomarker and neuroimaging assessments limits mechanistic interpretation. This study evaluated clinical and psychological outcomes using validated scales, but it did not directly measure neurotransmitters such as 5-HT or GABA, inflammatory factors such as IL-6 or TNF-α, neuroplasticity markers such as BDNF, cortisol levels, or brain network activity. Therefore, any discussion of neuroendocrine, inflammatory, neuroplastic, or brain-network mechanisms remains speculative and should be interpreted with caution. Future prospective studies could integrate blood biomarker analysis, neurophysiological assessments, or functional neuroimaging to further explore the potential biological mechanisms underlying the observed associations.

Finally, the balance between standardization and personalization in the intervention protocol requires further optimization. Although this study established structured EFT and psychological nursing procedures, the potential influence of patient individual differences (e.g., age, cultural background, disease stage) on observed psychological outcomes was not adequately explored. Introducing mobile health (mHealth) technologies, such as AI-based emotion monitoring apps and personalized intervention delivery systems, may further enhance the protocol's precision and accessibility.

## 5 Conclusion

Using a retrospective cohort design, this study examined the association between EFT combined with psychological nursing and psychological state and quality of life among breast cancer patients undergoing chemotherapy. The findings indicated that patients who received the combined intervention had lower anticipatory grief, anxiety, depression, and cancer-related fatigue scores, as well as higher hope and quality-of-life scores and more adaptive coping patterns, compared with patients who received conventional nursing. In addition, receipt of the combined intervention was associated with lower cancer-related fatigue and better quality-of-life scores. This study provides real-world evidence for psychological nursing practice in breast cancer patients undergoing chemotherapy. Compared to single-modality pharmacological interventions or conventional psychotherapy, EFT combined with psychological nursing offers advantages of being non-invasive, highly accessible, and demonstrating good patient compliance, supporting its further evaluation and potential application in oncology specialty hospitals and community medical centers. Future research should further evaluate the long-term clinical associations and potential biological mechanisms of the combined intervention through multicenter randomized controlled trials, long-term follow-up, and biomarker or neurophysiological assessments, while also exploring the application potential of mobile health technologies in protocol optimization.

In summary, EFT combined with psychological nursing may be considered a promising psychological nursing approach for breast cancer patients undergoing chemotherapy, but its causal effectiveness should be further confirmed through prospective, multicenter randomized controlled trials. Its clinical application is expected to improve patients' treatment experience and enhance quality of life, not only providing a new intervention model for breast cancer psychological nursing but also offering empirical evidence for constructing multimodal psychological intervention protocols in the field of psycho-oncology.

## Data Availability

The original contributions presented in the study are included in the article/supplementary material, further inquiries can be directed to the corresponding author.
